# Higher Adherence to Healthy Lifestyle Score Is Associated with Lower Odds of Non-Alcoholic Fatty Liver Disease

**DOI:** 10.3390/nu14214462

**Published:** 2022-10-24

**Authors:** Yu Zhu, Hu Yang, Shaoxian Liang, Honghua Zhang, Yufeng Mo, Songxian Rao, Yaozong Zhang, Zhuang Zhang, Weiqiang Wang, Wanshui Yang

**Affiliations:** 1 Department of Nutrition, School of Public Health, Anhui Medical University, Hefei 230032, China; 2 School of Public Health, Wannan Medical College, Wuhu 241002, China; 3 Department of General Practice, Suzhou Hospital of Anhui Medical University, Suzhou 234000, China

**Keywords:** cross-sectional study, healthy lifestyle, non-alcoholic fatty liver disease, liver function parameters, vibration-controlled transient elastography

## Abstract

Growing evidence supports that individual lifestyle factors contribute to the development of non-alcoholic fatty liver disease (NAFLD) without considering the coexistence and synergistic effect of lifestyle factors. Our aim is to derive a healthy lifestyle score (HLS) and estimate its association with NAFLD. In this nationwide cross-sectional study, we derived a five-item HLS including dietary pattern, body mass index, physical activity, cigarette smoking, and sleep duration. NAFLD and clinically significant fibrosis (CSF) were assessed based on vibration-controlled transient elastography (VCTE). Liver function parameters were also tested. Multivariable logistic and linear regressions were applied to investigate the association between HLS and liver diseases. Of the 3893 participants with VCTE examination, approximately 14.1% of participants possessed zero or one healthy lifestyle, 62.5% possessed two or three healthy lifestyles, and 23.4% possessed four or five healthy lifestyles. Compared with participants with a low HLS (0–1 score), the adjusted odds ratios and 95% confidence intervals for those with a high HLS (4–5 score) were 0.25 (0.19~0.33, *P_trend_* < 0.001) for NAFLD and 0.30 (0.18~0.50, *P_trend_* < 0.001) for CSF. HLS was positively associated with albumin, total protein, and total bilirubin (all *P_trend_* ≤ 0.001), and was inversely associated with globulin, alanine aminotransferase, and gamma-glutamyl transaminase (all *P_trend_* ≤ 0.003). Higher adherence to HLS is associated with lower odds of NAFLD and CSF and may improve liver function. Strategies for the promotion of a healthy lifestyle should be considered as part of NAFLD prevention.

## 1. Introduction

Non-alcoholic fatty liver disease (NAFLD), a condition characterized by the accumulation of triglycerides and other fats in liver cells in the absence of heavy drinking and secondary causes of liver disease, encompasses a continued spectrum of liver diseases ranging from simple steatosis without inflammation to advanced steatohepatitis, fibrosis, cirrhosis, and eventually hepatocellular carcinoma [1,2]. NAFLD has become the most prominent cause of chronic liver diseases worldwide, which affects up to 32% of the world’s population [3]. A study utilizing U.S. National Health and Nutrition Examination Surveys (NHANES) assessed the shift in the burden of different liver disease etiologies over the past three decades in the U.S. and reported that only the prevalence of NAFLD was growing, paralleling to the increase of obesity and diabetes [4].

Previous studies suggested that several lifestyle factors including obesity [5], tobacco smoking [6], short sleep duration [7], lack of physical activity [8], and an unhealthy diet [9], were associated with the risk of developing NAFLD. However, most studies merely focused on the association of individual lifestyle factors with NAFLD, which ignored the coexistence and synergistic effect of overall lifestyle factors. Therefore, it is essential to construct a comprehensive measure of these relevant lifestyle constituents. Prior studies have created a healthy lifestyle score (HLS) by summing the score of each lifestyle and have reported that greater adherence to a healthy lifestyle was associated with a lower risk of hypertension [10], liver cancer [11], total mortality [12,13], and cardiovascular disease-specific mortality [13]. To the best of our knowledge, only two cross-sectional studies have investigated the association between HLS and fatty liver disease (FLD) [14,15]. One study among 354 participants aged 48–77 years from Germany consisting largely of Caucasians, showed that a higher HLS was associated with lower odds of FLD determined by magnetic resonance imaging (MRI) [14]. Another study of a community-based sample of 2981 participants aged 40–75 years in China, reported that a higher HLS was associated with a lower prevalence of NAFLD determined by abdominal ultrasonography [15]. While no studies have examined the association between HLS and the risk of hepatic fibrosis. In addition, the narrow age groups in the two studies [14,15] and the sample of mainly Caucasian ethnicity in the first study [14] may limit the generalizability of the conclusions.

To add more evidence, we investigated the cross-sectional association between adherence to HLS and the odds of NAFLD and hepatic fibrosis assessed by vibration-controlled transient elastography (VCTE) in a nationally representative sample of U.S. adults aged 18 years or older. We also investigated the association between adherence to HLS and liver function parameters.

## 2. Methods

### 2.1. Study Population

In this study, participants were selected from the 2017 to 2018 cycle of the U.S. NHANES, which is a cross-sectional survey conducted by the National Center for Health Statistics (NCHS) of the Centers for Diseases Control and Prevention (CDC) in the United States. More details on the survey protocol of NHANES have been described elsewhere [16]. The study protocol was approved by NCHS Research Ethics Review Board (Protocol #2011-17; Protocol #2018-01) and written informed consent was obtained from all participants.

A total of 9254 participants were enrolled in the survey. Individuals aged ≥ 18 years old were included in this study. Individuals were excluded if they (i) had missing dietary data (*n* = 873) or an unreliable energy intake (defined as <600 or >3500 kcal/day for women; <800 or >4200 kcal/day for men, *n* = 221); (ii) had hepatitis B virus (HBV) infection (*n* = 18), hepatitis C virus (HCV) infection (*n* = 78), or excessive alcohol consumption (>3 drinks/d for men and >2 drinks/d for women, *n* = 116); (iii) did not have a valid VCTE detection (partial examination: *n* = 314; missing steatosis value: *n* = 1; not receiving VCTE detection: *n* = 255); (iv) had missing body mass index (BMI) data (*n* = 21), missing sleep data (*n* = 32), or missing physical activity data (*n* = 28, Supplementary Figure S1).

### 2.2. Definition of the HLS

A five-item HLS was derived in this study based on dietary patterns, BMI, physical activity, cigarette smoking, and sleep duration. Dietary intake was quantified via two 24-h dietary recalls. The first 24-h dietary recall was performed in person in the NHANES Mobile Examination Center (MEC), and a second 24-h dietary was conducted by telephone 3 to 10 days after the first recall. Dietary quality was assessed by the healthy eating index (HEI) scores. The HEI-2015 was calculated from two 24-h dietary recall data per participant. We defined a healthy dietary status as an HEI-2015 score in the top two-fifths of distribution.

Anthropometric measurement (height and weight) was conducted in MEC. BMI was calculated as weight (kg) divided by the square of the height (m), and the range from 18.5 to 24.9 was defined as a healthy status. Physical activity was measured using the global physical activity questionnaire through household interviews, which has been shown to have good reliability and validity in multiple populations [17,18]. A moderate-to-high level (≥500 metabolic equivalent task (MET)-minutes/week) of physical activity was defined as a healthy status. Cigarette smoking was obtained through household interviews with self-reported smoking of fewer than 100 cigarettes in life was defined as a healthy status. Sleep time was obtained using the Munich chronotype questionnaire through household interviews, a sleep duration of ≥7 h was defined as a healthy status.

Consistent with previous studies [13,19], we assigned 1 point for a healthy status and 0 points for an unhealthy status with respect to each item. The HLS was the sum of five-item scores, with a range from 0 to 5. A higher HLS denoted more adherence to a healthy lifestyle.

### 2.3. Assessments of Covariates

Standardized questionnaires were administrated through household interviews to collect demographic characteristics including age, sex, race/ethnicity, educational level, and income. Hypertension was defined if individuals (i) reported a history of hypertension; or (ii) had a systolic blood pressure (SBP) ≥ 140 mmHg; or (iii) had a diastolic blood pressure (DBP) ≥ 90 mmHg. Diabetes was defined if individuals (i) reported a diagnosis of diabetes; or (ii) had a hemoglobin A1c (HbA1c) level ≥ 6.5%; or (iii) had a fasting glucose level ≥ 126 mg/dL; or (iv) had a random glucose level ≥ 200 mg/dL.

### 2.4. Laboratory Assays and Liver Function Parameters

Liver function parameters, including serum albumin, globulin, total protein, total bilirubin, alanine aminotransferase (ALT), aspartate aminotransferase (AST), and gamma-glutamyl transaminase (GGT), were also obtained from participants. All laboratory procedures are described in detail elsewhere [20].

### 2.5. Ascertainments of NAFLD and Clinically Significant Fibrosis

VCTE was conducted by trained and certified technicians in MEC, using the FibroScan^®^ model 502 V2 Touch equipped with a medium or extra-large probe. Hepatic steatosis and fibrosis were assessed by controlled attenuation parameter (CAP) and liver stiffness measurement (LSM), respectively.

According to previous studies [21,22], a CAP score ≥ 285 dB/m was used to define NAFLD (CAP ≥ 285 dB/m) vs. non-NAFLD (CAP < 285 dB/m). Furthermore, a LSM score ≥ 8.6 kPa was used to define clinically significant fibrosis (CSF: LSM ≥ 8.6 kPa) vs. non-CSF (LSM < 8.6 kPa).

### 2.6. Statistical Analysis

Due to the limited number of individuals among the lowest or highest lifestyle score group, participants were grouped into low HLS (0–1 score), medium HLS (2–3 score), and high HLS (4–5 score). Continuous variables were presented as a mean (standard deviation) and categorical variables were presented as percentages. Characteristics of study participants were compared across three the HLS statuses using a one-way analysis of variance for continuous variables or a chi-square test for categorical variables or the Kruskal-Wallis test for ordinal variables.

We used multivariable logistic regression to evaluate the odds ratios (ORs) and 95% confidence intervals (CIs) for HLS status in relation to NAFLD and CSF. Model 1 did not adjust for the covariates and Model 2 was performed with adjustment for age (18–39, 40–59, ≥60), sex (male, female), race/ethnicity (non-Hispanic white, non-Hispanic black, and other races), education (less than high school, high school diploma, and more than high school), the ratio of family income to poverty (<1.30, 1.30–3.49, and ≥3.50), hypertension (yes, no), and diabetes (yes, no).

Given the limited number of CSF, we only estimated the stratified association between HLS and odds of NAFLD by age, sex, race/ethnicity, education level, the ratio of family income to poverty, hypertension, and diabetes. A Wald chi-square test was used to examine the cross-product terms between these variables and the HLS. Due to multiple tests being conducted, we used the Bonferroni correction to define the statistical significance as *p* < 0.007 (0.05/7) to account for the interaction effect.

Furthermore, considering departures from the normal distribution, all liver function parameters were natural logarithm transformed. Multivariable linear regression was performed to estimate the percentage change and 95% CIs for the associations between the HLS status and transformed liver function parameters, with adjustment for age, sex, race/ethnicity, education, the ratio of family income to poverty, hypertension, and diabetes. A two-sided *p*-value < 0.05 was considered to indicate statistical significance. All statistical analyses were performed using R software version 4.1.0 (University of Auckland, Auckland, New Zealand).

## 3. Results

### 3.1. Characteristics of Participants across HLS

A total of 3893 participants aged 18 to 80 years (mean age 49.3 ± 18.4 years) were included in the study. The prevalence was 36.9% (1437) for NAFLD and 7.7% (301) for CSF. Approximately 14.1% of participants possessed zero or one healthy lifestyle, whereas 62.5% possessed two or three healthy lifestyles, and 23.4% possessed four or five healthy lifestyles. Participants among the high HLS were more likely to be younger, female, and of other races, had a higher education level, a higher ratio of family income to poverty, and were less likely to have a history of hypertension and diabetes. In addition, we observed that the CAP, LSM, globulin, ALT, and GGT decreased with an increased HLS, whereas albumin, total protein, total bilirubin, and AST increased with an increased HLS (Table 1). Characteristics of participants according to the NAFLD and CSF phenotypes were shown in Table S1.

### 3.2. HLS and Odds of NAFLD and CSF

We observed a significant decline in the prevalence of NAFLD and CSF across HLS (Table 1). Compared with participants in low HLS, the adjusted ORs (95% CIs) of NAFLD for those in the high HLS group were 0.25 (0.19~0.33, *P_trend_* < 0.001). Similarly, a high HLS was associated with lower odds of CSF (OR = 0.30, 95% CI: 0.18~0.50, *P_trend_* < 0.001, Table 2).

When examining components in HLS, lower odds of NAFLD was observed among individuals with a healthy BMI level (OR = 0.13, 95% CI: 0.10~0.16), a healthy sleep level (OR = 0.73, 95% CI: 0.61~0.87), and a healthy HEI-2015 level (OR = 0.80, 95% CI: 0.69~0.94), whereas only a healthy BMI level (OR = 0.32, 95% CI: 0.20~0.50) and a healthy HEI-2015 level (OR = 0.66, 95% CI: 0.50~0.88) were associated with lower odds of CSF (Table 3).

**Figure 1:**
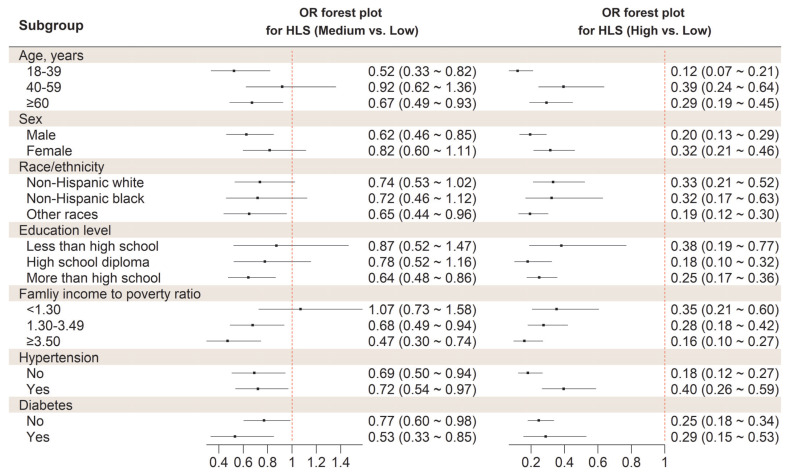
Stratified analysis on the association between HLS and odds of NAFLD. Abbreviations: HLS, Healthy lifestyle score; NAFLD, Non-alcoholic fatty liver diseases; OR, Odds ratio. The model was adjusted for age, sex, race/ethnicity, education level, the ratio of family income to poverty, hypertension, and diabetes except for variables examined in the figure.

In stratified analysis, we did not find any differential association of HLS with odds of NAFLD, according to age, sex, race/ethnicity, education level, ratio of family income to poverty, and diabetes (all *p* for interaction > 0.007). However, the inverse association between HLS and NAFLD appeared stronger in participants who were free of hypertension, compared with hypertension patients (*p* for interaction = 0.005) (Figure 1).

### 3.3. HLS and Liver Function Parameters

After multivariable adjustment, HLS was positively associated with albumin, total protein, total bilirubin, and AST, with a percentage difference of (High HLS vs. Low HLS) of 3.25% (95% CI: 2.35~4.16%, *P_trend_* < 0.001), 0.94% (95% CI: 0.24~1.64%, *P_trend_* = 0.001), 18.60% (95% CI: 11.50~26.16%, *P_trend_* < 0.001), and 6.60% (2.28~11.11%, *P_trend_* = 0.001), respectively. In contrast, HLS was inversely associated with globulin, ALT, and GGT, with a corresponding percentage difference of −1.95% (95% CI: −3.44~−0.42%, *P_trend_* = 0.003), −6.36% (95% CI: −11.8~−0.58%, *P_trend_* = 0.010), and -19.0% (95% CI: −24.7~−12.8%, *P_trend_* < 0.001, Table 4), respectively.

## 4. Discussion

In this large cross-sectional study, we found that greater adherence to HLS was associated with lower odds of NAFLD and CSF, where normal BMI was a key contributor. Furthermore, higher adherence to HLS appeared to be linked with improved hepatic parameters.

Several lifestyle factors, such as diet, physical activity, and smoking are postulated as important modifiable risk factors for chronic diseases. Numerous studies have linked lifestyle factors with chronic diseases and mortality, showing that adherence to a healthy lifestyle is beneficial for health and longevity [10,12,19]. These lifestyle factors often operate in a synergistic manner, which cannot be captured when studying each individually [23]. Instead of examining each lifestyle factor independently, a holistic approach integrating the whole lifestyle pattern was proposed. Despite divergent definitions of HLS, two cross-sectional studies have appraised the association between HLS and FLD [14,15] and yielded similar results to our current findings. One of the studies constructed a four-item HLS by incorporating smoking, waist circumference, physical activity, and diet [14], whereas a six-item HLS including smoking, BMI, physical activity, diet, sleep, and anxiety was built in the other study [15]. The components of three constructed HLS have considerable overlap, indicating that these key individual lifestyle factors play a crucial role in modulating health outcomes.

The two previously published studies did not investigate the association of HLS with liver function parameters. Liver diseases develop silently with no signs or symptoms until the late stages. In pre-terminal stages, liver chemistries, which are commonly ordered in comprehensive metabolic profiles, may be abnormal and contribute to the early detection of diseases. Consistent with the link between HLS and VCTE-determined NAFLD, most of these liver chemistries varied significantly across HLS. On the one hand, we observed elevated serum albumin, total protein, and total bilirubin among participants with high HLS. Albumin is exclusively synthesized by the liver, and low albumin levels may be a marker of advanced diseases in chronic liver inflammation or cirrhosis [24,25]. Bilirubin is the end product of the breakdown of red blood cells. A growing body of literature shows that higher bilirubin levels are inversely associated with NAFLD as well as metabolic syndrome [26,27], although the underlying mechanisms are not well elucidated. On the other hand, a decline in serum globulin, ALT, and GGT were observed in adults with high HLS. Globulin is a group of proteins synthesized mainly in the liver by the immune system and an increase in globulin may indicate inflammatory diseases [28]. ALT and GGT are released from damaged liver cells into the blood after hepatocellular injury or death [29,30], those are considered to serve as non-invasive biomarkers of liver injury.

Until now, no approved pharmacotherapy for NAFLD exists. Practice guidance advocates that lifestyle interventions mainly consisting of weight loss, exercise, and dietary change remain the primary approach for the management of NAFLD across the disease spectrum [31,32]. NAFLD is strongly associated with obesity with a prevalence of up to 80% in obese adults, and 16% in adults with a normal BMI [33]. In this study, BMI was observed as a stronger contributor to the development of NAFLD and CSF among the five lifestyles. Obesity contributes to NAFLD by either increasing the synthesis of triglycerides in the liver or increasing the flow of fatty acids from adipose tissue to the liver [34]. Slow and controlled weight loss is the key to the improvement in the histopathological features of non-alcoholic steatohepatitis [32]. A meta-analysis of eight randomized controlled trials assessed the effect of weight loss on NAFLD [35] and suggested that losing at least 5% of body weight could improve hepatic steatosis, whereas a ≥7% body weight reduction was associated with an improvement in the NAFLD activity score. The findings from a prospective study of 293 patients with histologically proven non-alcoholic steatohepatitis indicated that greater than 10% weight loss was able to ameliorate portal inflammation and fibrosis [36].

Some dietary patterns have emerged as a novel approach to examining the association between diet and the risk of chronic liver diseases. Our recent findings [37] together with a hospital-based case-control study [38] performed in Iran consistently supported that a higher dietary insulinaemic potential was linked with an increased prevalence of hepatic steatosis and fibrosis. In addition, the dietary inflammatory index [39], and dietary approaches to stop hypertension [40] were also shown to be associated with NAFLD. In the present study, we calculated HEI-2015 to assess diet quality. The HEI-2015 was originally designed to evaluate diet for compliance with the Dietary Guidelines for Americans (DGA) 2015–2020 [41]. DGA encourages increasing the consumption of fruits, vegetables, whole grains, high-protein food from seafood, lean meats, poultry, eggs, and nuts, and limiting the intake of saturated fats, trans fats, and added sugars. An energy-restricted diet with a low calorie (1200–1600 kcal/d), low fat (less than 10% of energy from saturated fats), low carbohydrate diet (less than 50% of energy) is recommended for NAFLD patients [42].

Our findings indicated that lack of sleep was another contributor to NAFLD. Several epidemiological studies have examined the relationship between sleep duration and NAFLD and have demonstrated short sleep duration was an important risk factor for the onset of NAFLD [7,43]. This association may be driven by systemic inflammation and metabolic disorder-related pathways, including insulin resistance and adipose dysfunction [44]. Furthermore, previous studies have shown that cigarette smoking [45] and lack of physical activity [46] were associated with an increased risk of NAFLD and CSF. Although our study did not support a significant association between having healthy smoking and physical activity levels and the presence of NAFLD and CSF, most associations were inverse and thus were suggestive of possible benefits of having healthy smoking and physical activity levels. These inconsistent findings might be due to chance or no consensus on the definition of a harmful level of cigarette smoking and physical activity.

We found a significant interaction between HLS and hypertension on the odds of developing NAFLD, whereby adherence to HLS yielded a stronger health benefit against NAFLD among participants free of hypertension. Existing studies have shown a comorbidity phenomenon of hypertension and NAFLD. It is not clear whether this simply reflects shared risk factors, or whether hypertension, *per se*, increases the risks of NAFLD [47]. The prevalence of NAFLD increased progressively from 27.2% at optimal blood pressure (SBP < 120 mmHg and DBP < 80 mmHg) to 59.6% in participants with hypertension. Similarly, the prevalence of advanced fibrosis increased from optimal blood pressure to hypertension at 0.8% and 5.4%, respectively [48]. Therefore, this limited the health benefit of HLS among participants with hypertension.

Several limitations should be addressed. First, self-reported cigarette smoking, sleep duration, and physical activity as well as other covariates from questionnaires had measurement errors, although several methods were used to reduce measurement error in the investigation. Second, dietary information was collected based on 24-h recall. Although two 24-h recalls were conducted, it was difficult to capture the long-term dietary habits. Third, the cross-sectional design of the current study did not allow the determination of causality. Findings from our study need to be confirmed by prospective cohort studies and clinical trials in the future.

## 5. Conclusions

In conclusion, our findings pointed to the important association of HLS comprised of five favorable lifestyle factors with NAFLD and CSF in a representative sample of U.S. adults. With the increasing number of favorable lifestyle factors, the odds of NAFLD and CSF decreased. Strategies for the promotion of a healthy lifestyle should be considered as part of NAFLD prevention in adults.

## Figures and Tables

**Table 1:** The characteristics of participants according to status of healthy lifestyle score ^†^.

Characteristics	Overall	Healthy Lifestyle Score (HLS) ^‡^			*p*
		Low HLS	Medium HLS	High HLS	
No. of Participants	3893	547 (14.05)	2434 (62.52)	912 (23.43)	
Age (%)					<0.001
18–39	1330 (34.16)	123 (22.49)	817 (33.57)	390 (42.76)	
40–59	1172 (30.11)	172 (31.44)	750 (30.81)	250 (27.41)	
≥60	1391 (35.73)	252 (46.07)	867 (35.62)	272 (29.82)	
Sex (Female, %)	2030 (52.14)	259 (47.35)	1226 (50.37)	545 (59.76)	<0.001
Race (%)					
Non-Hispanic white	1360 (34.93)	230 (42.05)	860 (35.33)	270 (29.61)	
Non-Hispanic black	876 (22.50)	152 (27.79)	588 (24.16)	136 (14.91)	
Other races	1657 (42.56)	165 (30.16)	986 (40.51)	506 (55.48)	
Education (%)					<0.001
Less than high school	691 (17.78)	103 (18.83)	479 (19.71)	109 (11.99)	
High school diploma	954 (24.55)	160 (29.25)	610 (25.10)	184 (20.24)	
More than high school	2241 (57.67)	284 (51.92)	1341 (55.19)	616 (67.77)	
Family income to poverty ratio (%)					<0.001
<1.30	956 (27.74)	164 (34.24)	599 (27.91)	193 (23.51)	
1.30–3.49	1389 (40.31)	209 (43.63)	902 (42.03)	278 (33.86)	
≥3.50	1101 (31.95)	106 (22.13)	645 (30.06)	350 (42.63)	
Hypertension (%)	1592 (41.58)	291 (54.39)	1035 (43.23)	266 (29.56)	<0.001
Diabetes (%)	732 (18.80)	143 (26.14)	484 (19.88)	105 (11.51)	<0.001
NAFLD (%)	1437 (36.91)	270 (49.36)	989 (40.63)	178 (19.52)	<0.001
CSF (%)	301 (7.73)	64 (11.70)	210 (8.63)	27 (2.96)	<0.001
Steatosis (CAP, dB/m)	263.29 ± 62.91	282.21 ± 61.94	269.33 ± 62.63	235.81 ± 55.37	<0.001
Fibrosis (LSM, kPa)	5.70 ± 4.51	6.38 ± 5.73	5.84 ± 4.75	4.92 ± 2.43	<0.001
Albumin (g/dL)	4.08 ± 0.32	4.00 ± 0.35	4.06 ± 0.31	4.17 ± 0.31	<0.001
Globulin (g/dL)	3.07 ± 0.42	3.09 ± 0.45	3.09 ± 0.42	3.03 ± 0.41	0.004
Total protein (g/dL)	7.15 ± 0.43	7.09 ± 0.43	7.15 ± 0.43	7.20 ± 0.43	<0.001
Total bilirubin (mg/dL)	0.46 ± 0.28	0.43 ± 0.24	0.45 ± 0.26	0.51 ± 0.33	<0.001
ALT (IU/L)	22.04 ± 17.16	21.49 ± 13.55	23.02 ± 17.59	19.77 ± 17.70	<0.001
AST (IU/L)	21.41 ± 11.15	20.29 ± 8.64	21.79 ± 12.01	21.08 ± 9.99	0.013
GGT (IU/L)	29.87 ± 41.27	35.41 ± 53.22	31.18 ± 43.53	23.07 ± 21.24	<0.001

Abbreviations: ALT, Alanine aminotransferase; AST, Aspartate aminotransferase; CAP, Controlled attenuation parameter; CSF, clinically significant fibrosis; GGT, Gamma-glutamyl transaminase; HLS, Healthy lifestyle score; LSM, Liver stiffness measurement; NAFLD, Non-alcoholic fatty liver diseases; NHANES, U.S. National Health and Nutrition Examination Survey. ^†^ Values were presented as mean ± SD or percentages. ^‡^ Low HLS: 0–1 score; Medium HLS: 2–3 score; High HLS: 4–5 score.

**Table 2:** Odds ratios and 95% confidence intervals of NAFLD and CSF according to healthy lifestyle scores.

Liver Disease	Healthy Lifestyle Score (HLS) ^†^			Per 1 Score	*P_trend_* ^¶^
	Low HLS	Medium HLS	High HLS		
NAFLD					
No. of cases/participants	270/547	989/2434	178/912		
Model 1 ^‡^	1	0.70 (0.58~0.85)	0.25 (0.20~0.31)	0.66 (0.62~0.70)	<0.001
Model 2 ^§^	1	0.71 (0.58~0.89)	0.25 (0.19~0.33)	0.66 (0.61~0.71)	<0.001
CSF					
No. of cases/participants	64/547	210/2434	27/912		
Model 1 ^‡^	1	0.71 (0.53~0.96)	0.23 (0.15~0.37)	0.69 (0.62~0.77)	<0.001
Model 2 ^§^	1	0.81 (0.58~1.12)	0.30 (0.18~0.50)	0.76 (0.67~0.86)	<0.001

Abbreviations: CSF, clinically significant fibrosis; NAFLD, Non-alcoholic fatty liver diseases. ^†^ Low HLS: 0–1 score; Medium HLS: 2–3 score; High HLS: 4–5 score. ^‡^ Model 1 did not adjust for the covariates. ^§^ Model 2 was adjusted for age (18–39, 40–59, ≥60), sex (male, female), race/ethnicity (non-Hispanic white, non-Hispanic black, and other races), education (less than high school, high school diploma, and more than high school), the ratio of family income to poverty (<1.30, 1.30–3.49, and ≥3.50), hypertension (yes, no), and diabetes (yes, no). ^¶^ The trend test was performed by using HLS as a continuous variable in the models.

**Table 3:** Odds ratios and 95% confidence intervals of NAFLD and CSF by individual lifestyle factors ^†^.

Components of HLS	NAFLD Phenotype		CSF Phenotype	
	OR and 95% CI	*p*	OR and 95% CI	*p*
BMI score (1 vs. 0)	0.13 (0.10~0.16)	<0.001	0.32 (0.20~0.50)	<0.001
Smoking score (1 vs. 0)	0.93 (0.79~1.09)	0.377	0.97 (0.74~1.28)	0.835
Sleep score (1 vs. 0)	0.73 (0.61~0.87)	<0.001	0.90 (0.67~1.21)	0.469
Physical activity score (1 vs. 0)	0.87 (0.74~1.02)	0.087	0.83 (0.63~1.08)	0.169
HEI-2015 score (1 vs. 0)	0.80 (0.69~0.94)	0.006	0.66 (0.50~0.88)	0.004

Abbreviations: BMI, Body mass index; CSF, clinically significant fibrosis; HEI, Healthy eating index; NAFLD, Non-alcoholic fatty liver diseases. ^†^ Model was adjusted for age (18–39, 40–59, ≥60), sex (male, female), race/ethnicity (non-Hispanic white, non-Hispanic black, other races), education (less than high school, high school diploma, more than high school), the ratio of family income to poverty (<1.30, 1.30–3.49, ≥3.50), hypertension (yes, no), and diabetes (yes, no).

**Table 4:** Percentage change (%) and 95% confidence intervals for the associations between the healthy lifestyle score and liver function parameters ^†^.

Parameters	Healthy Lifestyle Score (HLS) ^§^			Per 1 Score	*P_trend_* ^¶^
	Low HLS	Medium HLS	High HLS		
Albumin ^‡^	0	0.89 (0.13~1.64)	3.25 (2.35~4.16)	1.04 (0.80~1.28)	<0.001
Globulin ^‡^	0	−0.48 (−1.78~0.84)	−1.95 (−3.44~−0.42)	−0.63 (−1.05~−0.22)	0.003
Total protein ^‡^	0	0.20 (−0.39~0.79)	0.94 (0.24~1.64)	0.31 (0.13~0.50)	0.001
Total bilirubin ^‡^	0	4.36 (−1.00~10.02)	18.60 (11.50~26.16)	5.89 (4.13~7.68)	<0.001
ALT ^‡^	0	3.59 (−1.57~9.03)	−6.36 (−11.8~−0.58)	−2.11 (−3.69~−0.51)	0.010
AST ^‡^	0	4.82 (1.17~8.59)	6.60 (2.28~11.11)	1.88 (0.74~3.03)	0.001
GGT ^‡^	0	−5.56 (−11.3~0.58)	−19.0 (−24.7~−12.8)	−6.30 (−8.16~−4.41)	<0.001

Abbreviations: ALT, Alanine aminotransferase; AST, Aspartate aminotransferase; GGT, Gamma-glutamyl transaminase. ^†^ Percentage change was calculated as (e^β^ − 1) ∗ 100% based on multivariable linear regression with adjustment for age, sex, race/ethnicity, education, ratio of family income to poverty, hypertension, and diabetes. ^‡^ Liver function parameters were natural logarithm transformed. ^§^ Low HLS: 0–1 score; Medium HLS: 2–3 score; High HLS: 4–5 score. ^¶^ Linear trend test was performed using HLS as a continuous variable in the models.

## Data Availability

Data described in the manuscript, code book, and analytic code will be made publicly and freely available without restriction (https://www.cdc.gov/nchs/nhanes/index.htm, accessed on 11 April 2021).

## Supplementary Materials

The following supporting information can be downloaded at: https://www.mdpi.com/article/10.3390/nu14214462/s1, Figure S1: Flow chart of the study participants from the 2017–2018 cycle of the NHANES; Table S1: The characteristics of participants according to NAFLD and CSF phenotypes.
