# Supplementary material for: Higher Adherence to Healthy Lifestyle Score Is Associated with Lower Odds of Non-Alcoholic Fatty Liver Disease
Source: Nutrients. 2022 Oct 24;14(21):4462. doi: 10.3390/nu14214462 (PMC9657000; doi:10.3390/nu14214462)
Supplement: Supplementary file 1 [file nutrients-14-04462-s001.zip › nutrients-1952962-supplementary.pdf]

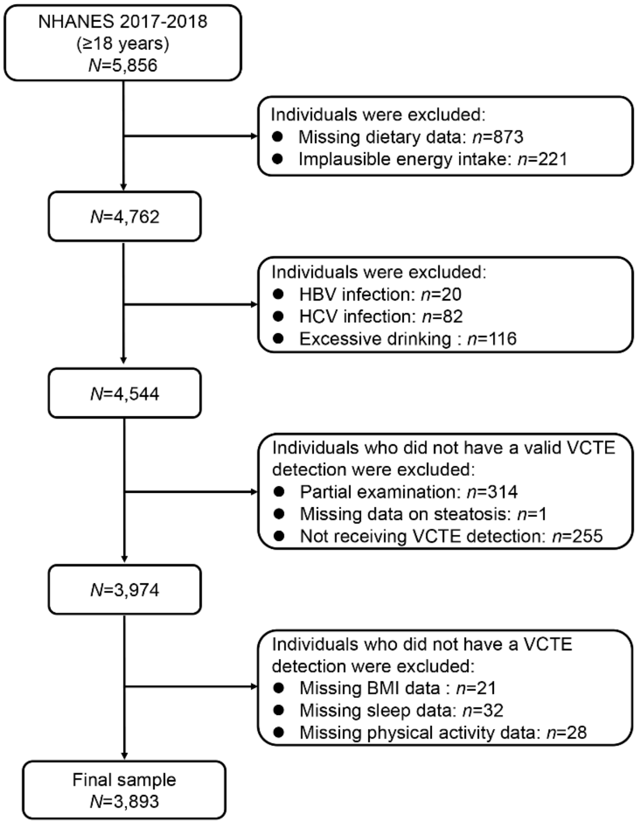

Figure S1. Flow chart of the study participants from the 2017-2018 cycle of the NHANES.
Abbreviations, BMI, Body mass index; HBV, Hepatitis B Virus; HCV, Hepatitis C Virus, NHANES, U.S. National Health and Nutrition Examination Survey; VCTE, Vibration-controlled transient elastography.

Table S1. The characteristics of participants according to NAFLD and CSF phenotypes †.

Characteristics	Overall	NAFLD phenotype			CSF phenotype		
		non-NAFLD	NAFLD	*P*	non-CSF	CSF	*P*
No. of Participants (%)	3,893	2,456(63.09)	1,437(36.91)		3,592(92.27)	301(7.73)	
Age (%)				<0.001			<0.001
18-39	1,330(34.16)	996(40.55)	334(23.24)		1,269(35.33)	61(20.27)	
40-59	1,172(30.11)	657(26.75)	515(35.84)		1,083(30.15)	89(29.57)	
≥60	1,391(35.73)	803(32.70)	588(40.92)		1,240(34.52)	151(50.17)	
Sex (Male, %)	1,863(47.86)	1,075(43.77)	788(54.84)	<0.001	1,688(46.99)	175(58.14)	<0.001
Race (%)				<0.001			0.197
Non-Hispanic white	1,360(34.93)	831(33.84)	529(36.81)		1,241(34.55)	119(39.53)	
Non-Hispanic black	876(22.50)	625(25.45)	251(17.47)		816(22.72)	60(19.93)	
Other races	1,657(42.56)	1,000(40.72)	657(45.72)		1,535(42.73)	122(40.53)	
Education (%)				0.198			0.011
Less than high school	691(17.78)	425(17.33)	266(18.56)		628(17.52)	63(20.93)	
High school diploma	954(24.55)	595(24.26)	359(25.05)		868(24.21)	86(28.57)	
More than high school	2,241(57.67)	1,433(58.42)	808(56.39)		2,089(58.27)	152(50.50)	
Family income to poverty ratio (%)				0.623			0.413
0-	956(27.74)	618(28.47)	338(26.51)		883(27.85)	73(26.55)	
1.3-	1,389(40.31)	855(39.38)	534(41.88)		1,262(39.80)	127(46.18)	
3.5-	1,101(31.95)	698(32.15)	403(31.61)		1,026(32.36)	75(27.27)	
Hypertension (%)	1,592(41.58)	823(34.09)	769(54.35)	<0.001	1,394(39.48)	198(66.44)	<0.001
Diabetes (%)	732(18.80)	271(11.03)	461(32.08)	<0.001	602(16.76)	130(43.19)	<0.001
Healthy Lifestyle Score (HLS, %) ‡				<0.001			<0.001
Low HLS	547(14.05)	277(11.28)	270(18.79)		483(13.45)	64(21.26)	
Medium HLS	2,434(62.52)	1,445(58.84)	989(68.82)		2,224(61.92)	210(69.77)	
High HLS	912(23.43)	734(29.89)	178(12.39)		885(24.64)	27(8.97)	
Albumin (g/dL)	4.08±0.32	4.09±0.32	4.05±0.32	<0.001	4.08±0.32	4.00±0.33	<0.001
Globulin (g/dL)	3.07±0.42	3.06±0.42	3.10±0.42	0.003	3.07±0.41	3.14±0.50	0.017
Total protein (g/dL)	7.15±0.43	7.15±0.43	7.15±0.42	0.813	7.15±0.43	7.14±0.46	0.586
Total bilirubin (mg/dL)	0.46±0.28	0.47±0.29	0.45±0.26	0.046	0.46±0.27	0.51±0.28	0.002
ALT (IU/L)	22.04±17.16	18.89±14.91	27.32±19.26	<0.001	21.21±15.65	31.85±27.92	<0.001
AST (IU/L)	21.41±11.15	20.43±10.25	23.06±12.35	<0.001	20.85±9.72	28.08±20.75	<0.001
GGT (IU/L)	29.87±41.27	24.81±37.44	38.36±45.76	<0.001	27.65±37.19	56.25±68.99	<0.001

Abbreviations: ALT, Alanine aminotransferase; AST, Aspartate aminotransferase; CSF, Clinically significant fibrosis; GGT, Gamma-glutamyl transaminase; NAFLD, Non-alcoholic fatty liver diseases; NHANES, U.S. National Health and Nutrition Examination Survey.

† Values were presented as mean±SD or percentages;

‡ Low HLS: 0-1 score; Medium HLS: 2-3 score; High HLS: 4-5 score.
